# Supplementary figures and images for: Temporal resolution of spike coding in feedforward networks with signal convergence and divergence
Source: PLoS Comput Biol. 2025 Apr 21;21(4):e1012971. doi: 10.1371/journal.pcbi.1012971 (PMC12021431; doi:10.1371/journal.pcbi.1012971)

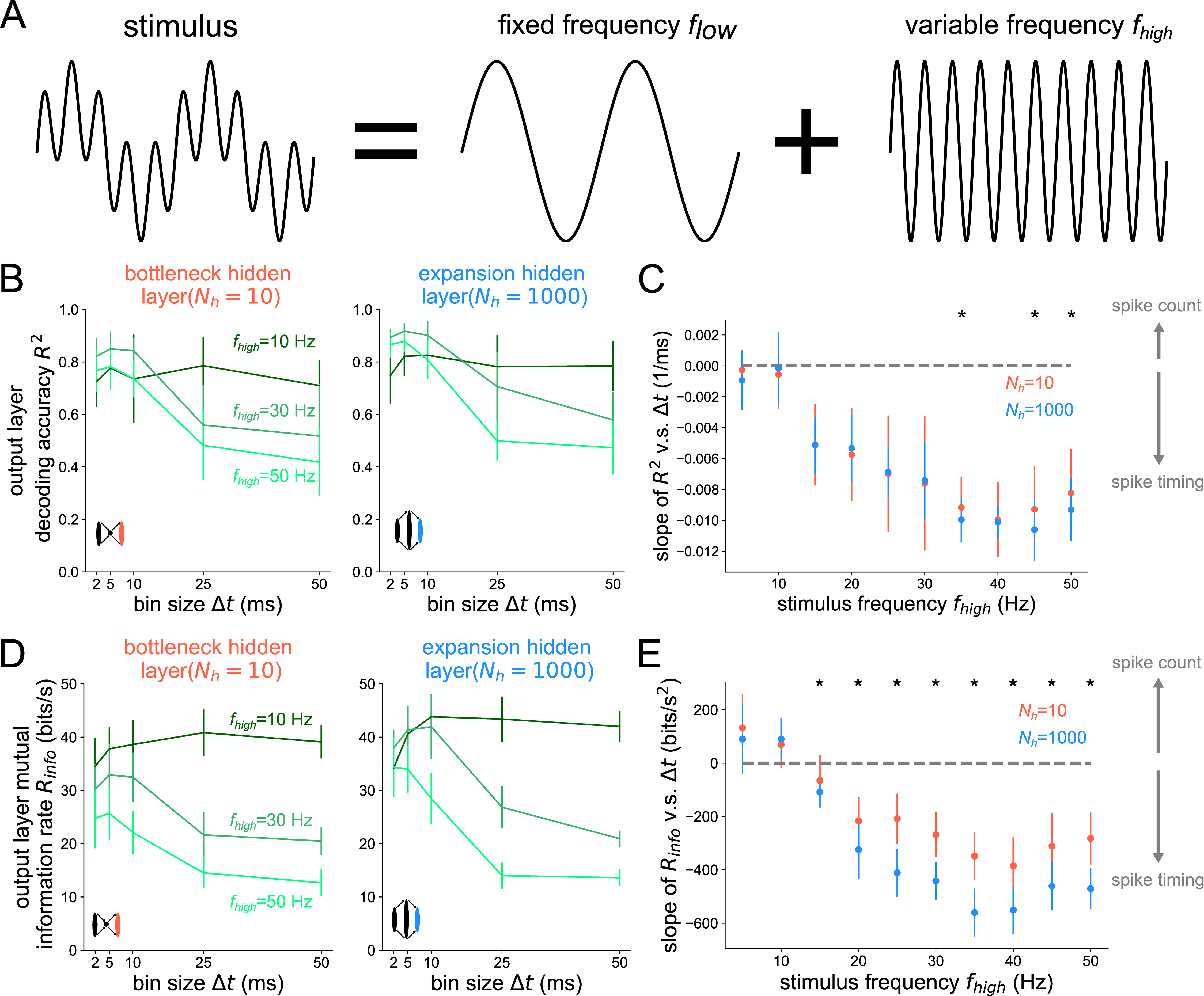

Supplement: S1 Fig — (TIF) [file pcbi.1012971.s001.tif]

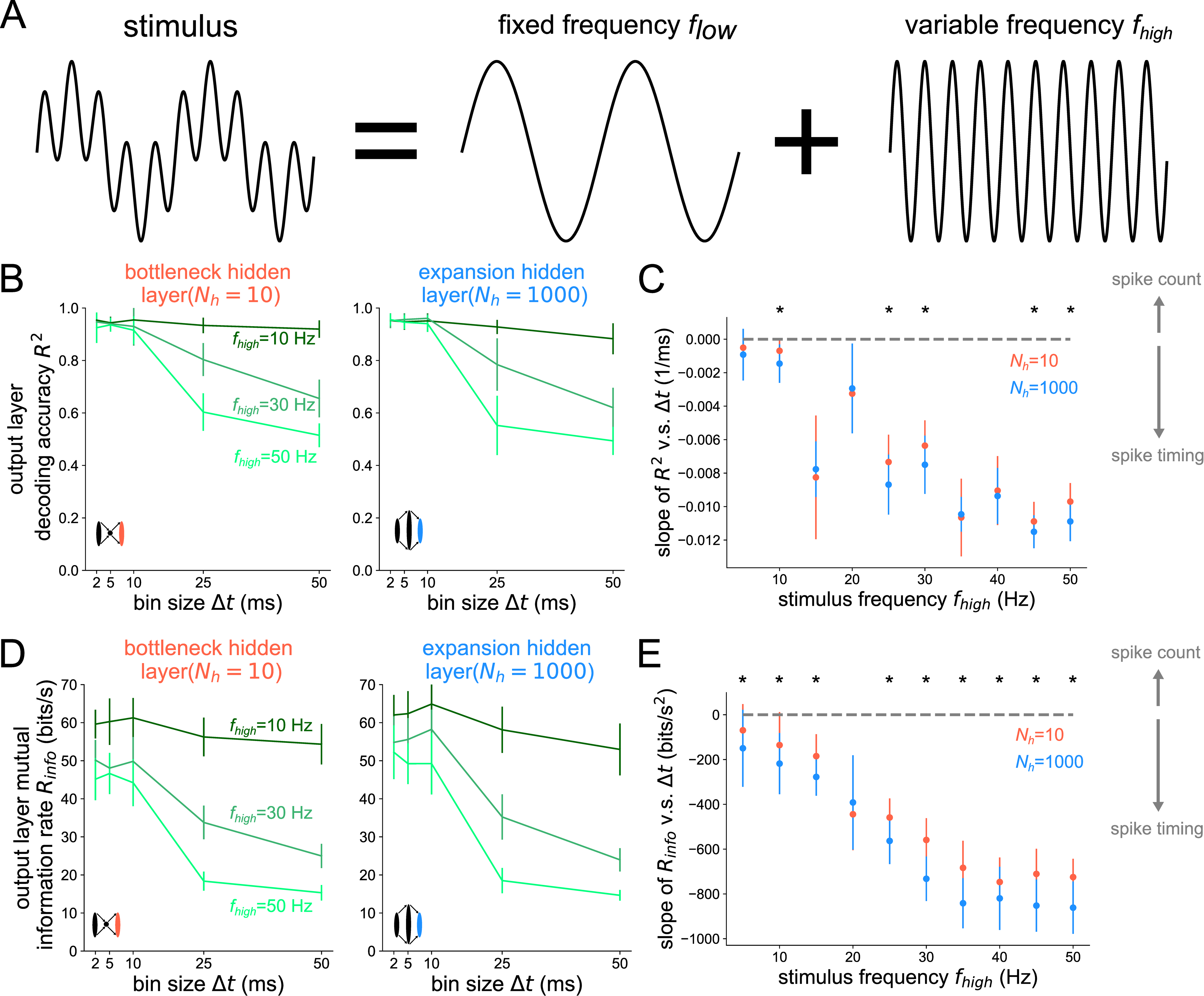

Supplement: S2 Fig — (TIF) [file pcbi.1012971.s002.tif]

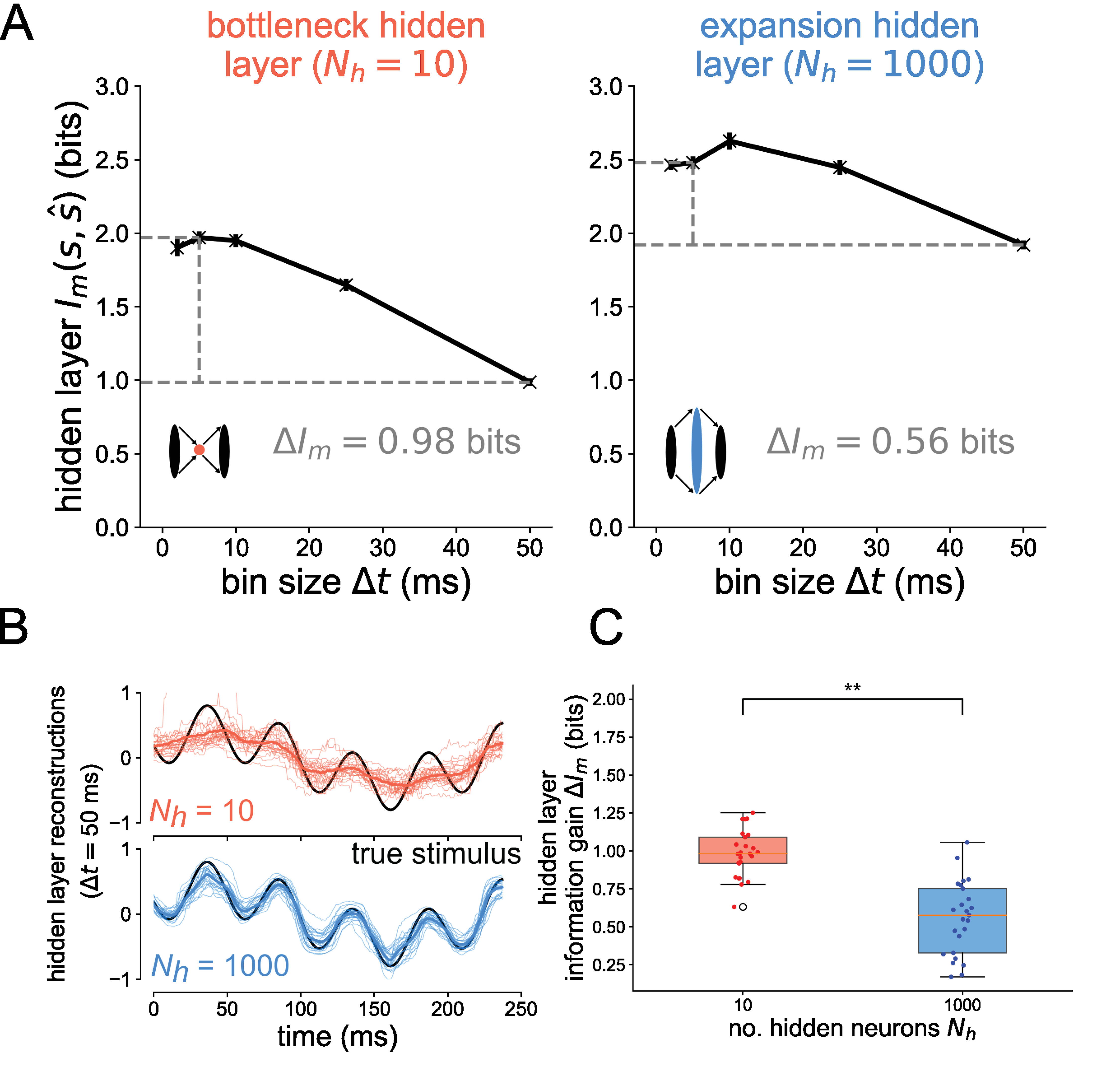

Supplement: S3 Fig — (TIF) [file pcbi.1012971.s003.tif]

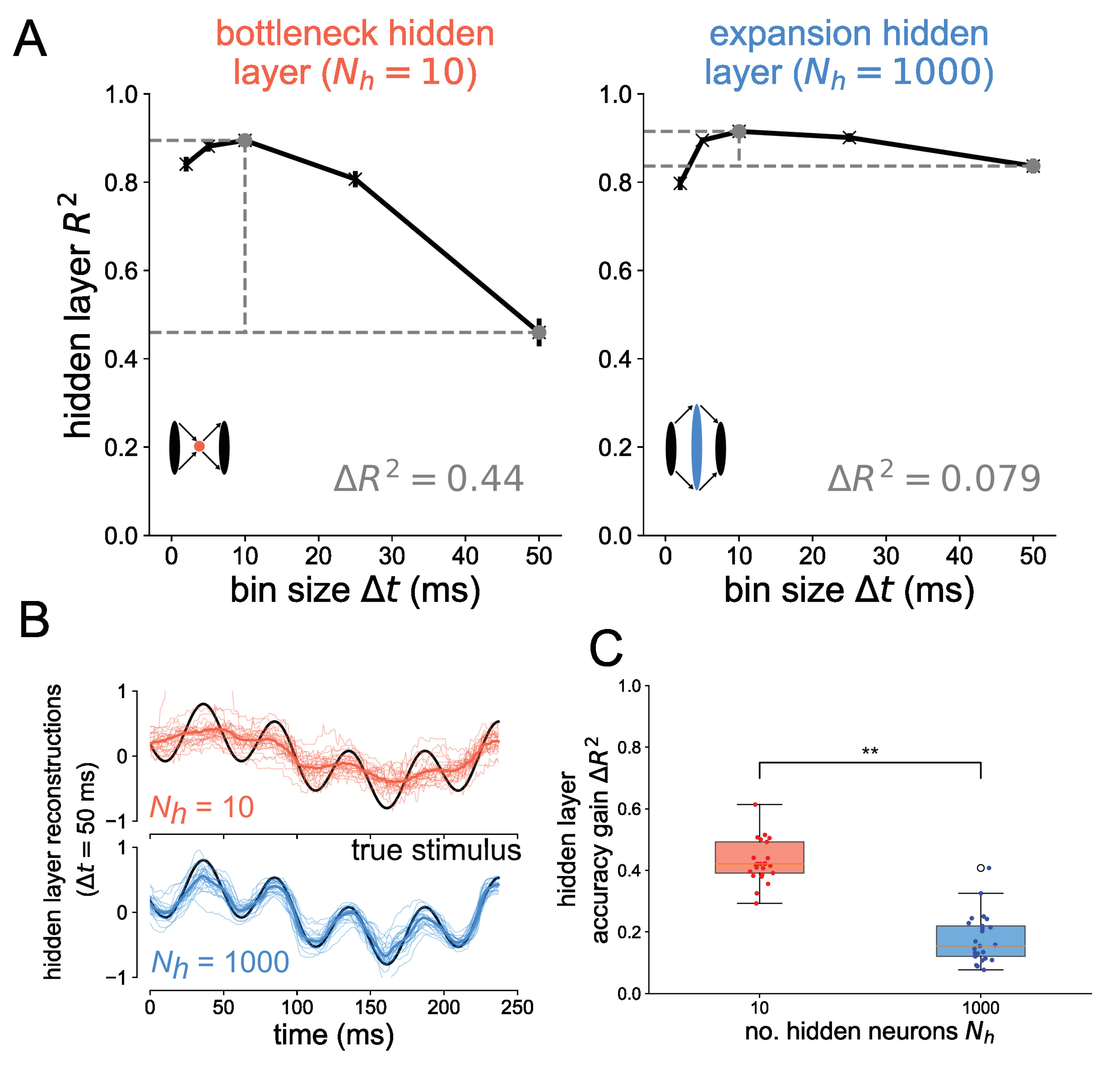

Supplement: S4 Fig — (TIF) [file pcbi.1012971.s004.tif]

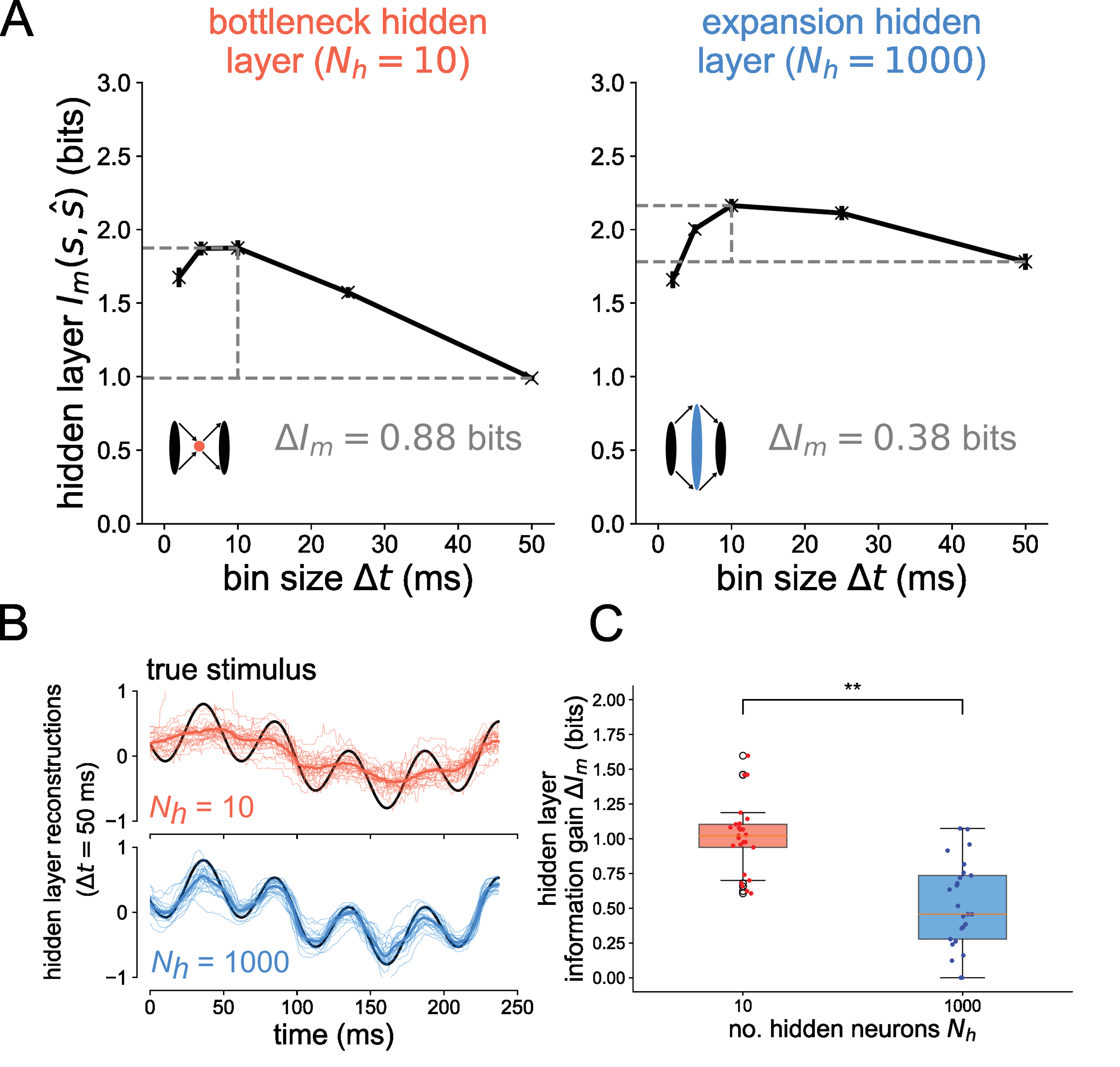

Supplement: S5 Fig — (TIF) [file pcbi.1012971.s005.tif]

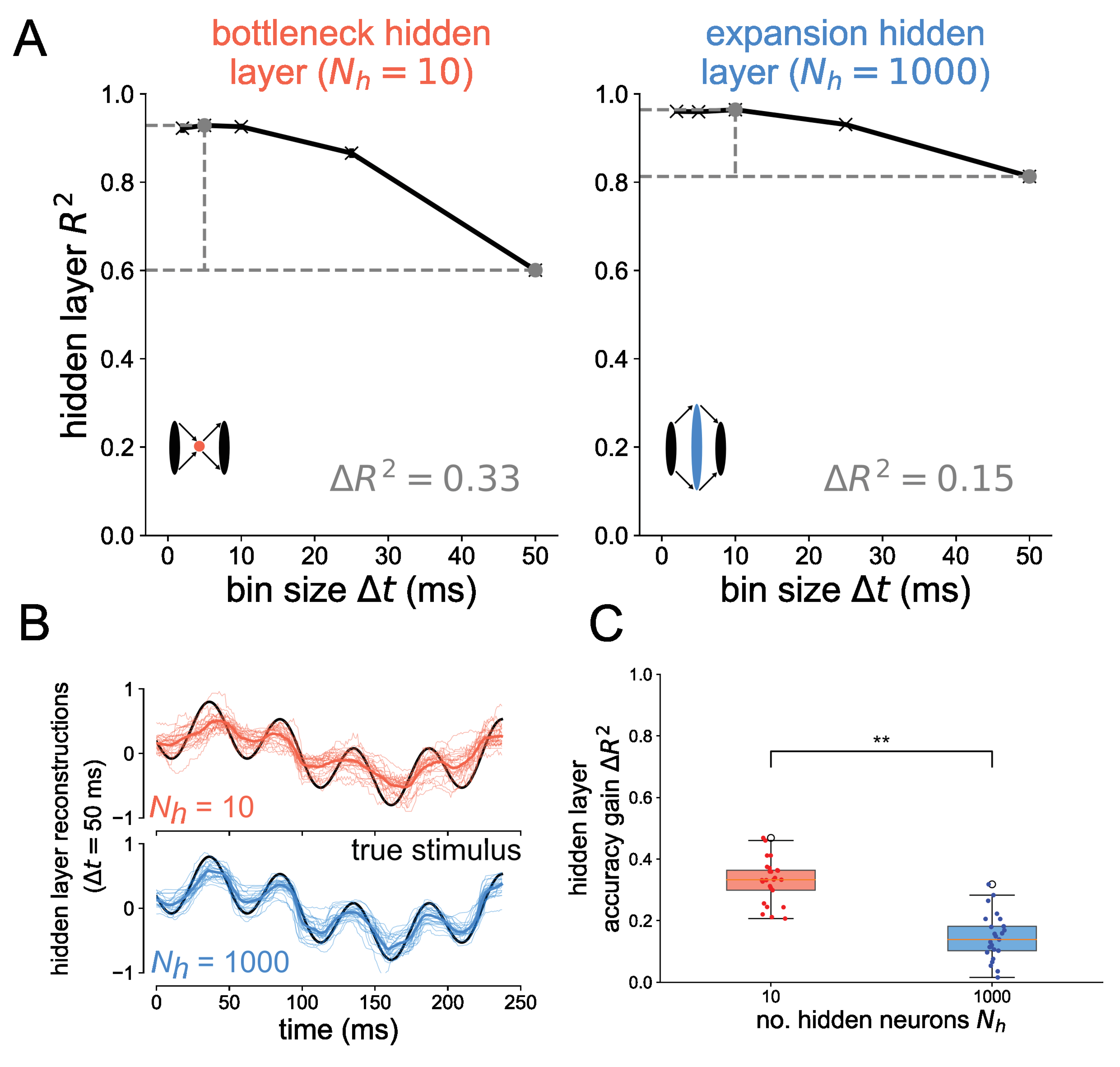

Supplement: S6 Fig — (TIF) [file pcbi.1012971.s006.tif]

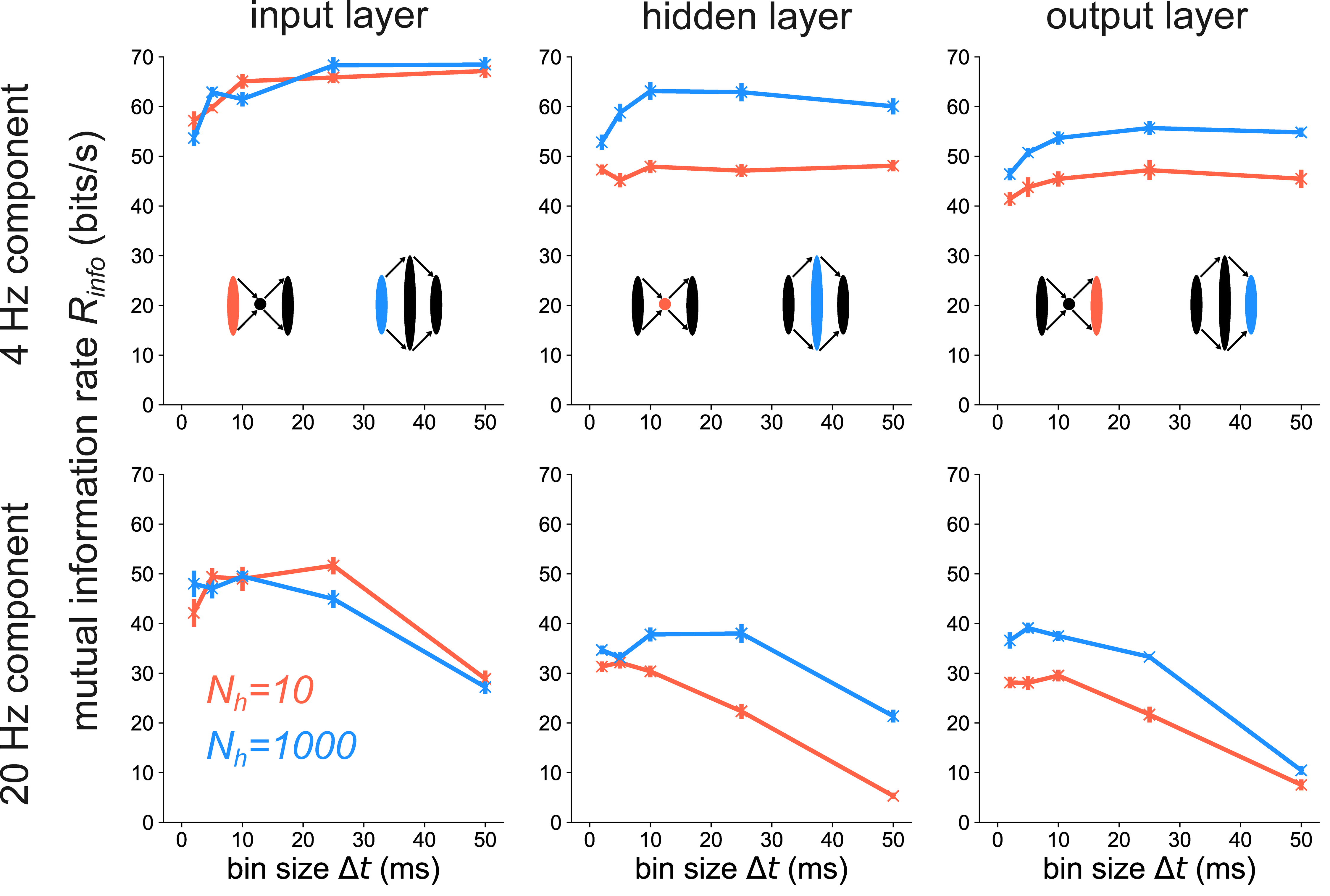

Supplement: S7 Fig — (TIF) [file pcbi.1012971.s007.tif]

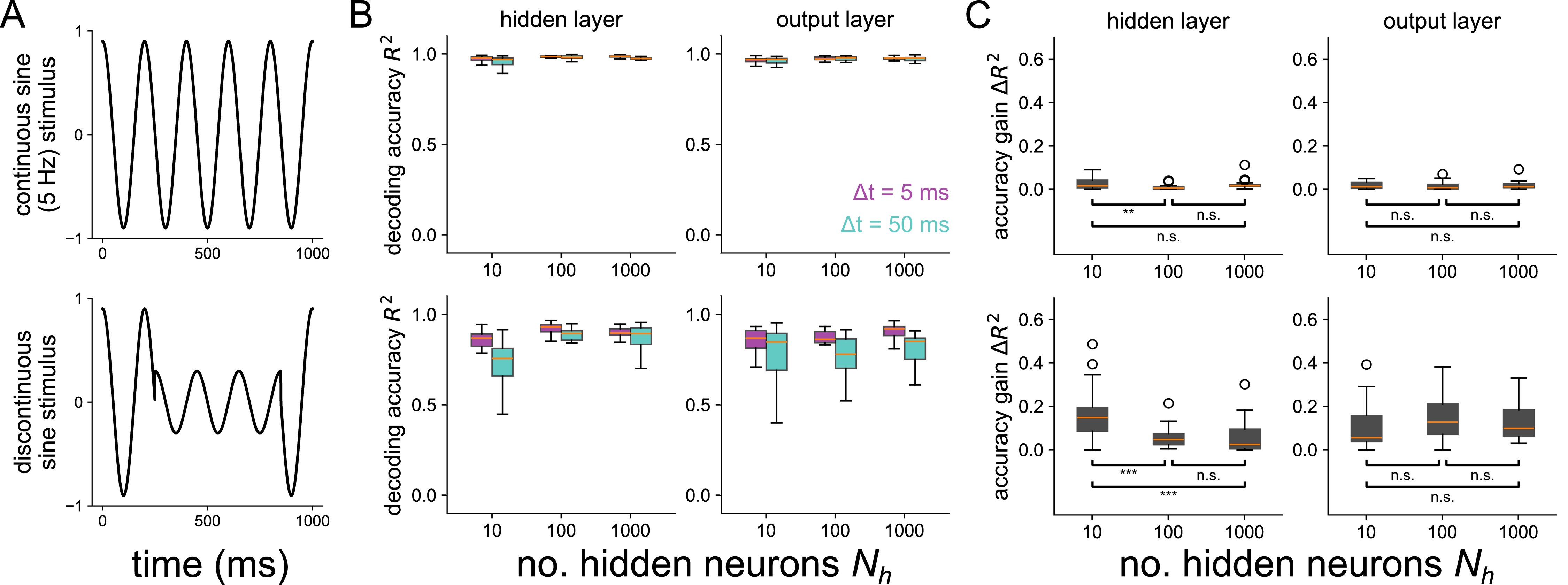

Supplement: S8 Fig — (TIF) [file pcbi.1012971.s008.tif]

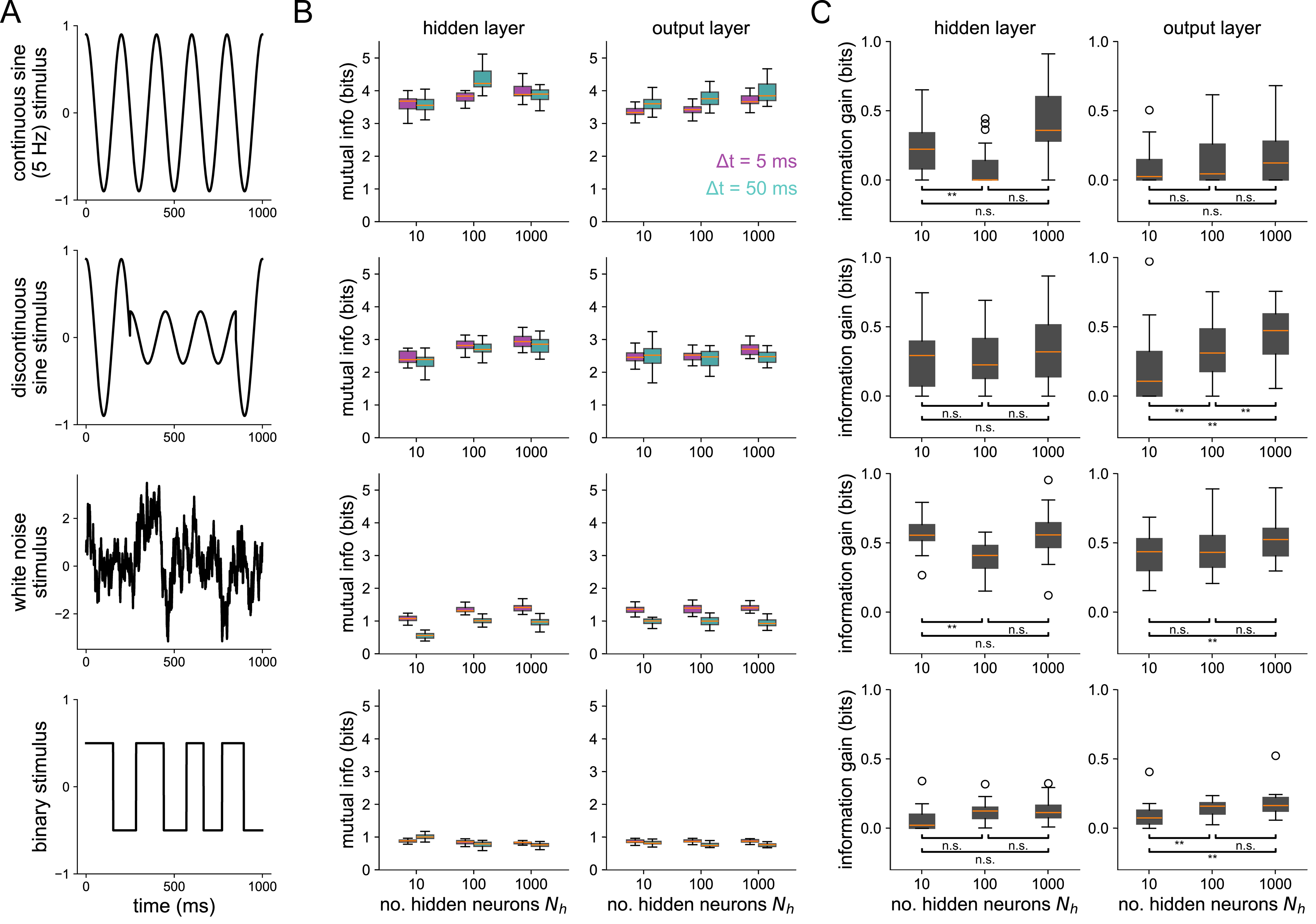

Supplement: S9 Fig — (TIF) [file pcbi.1012971.s009.tif]

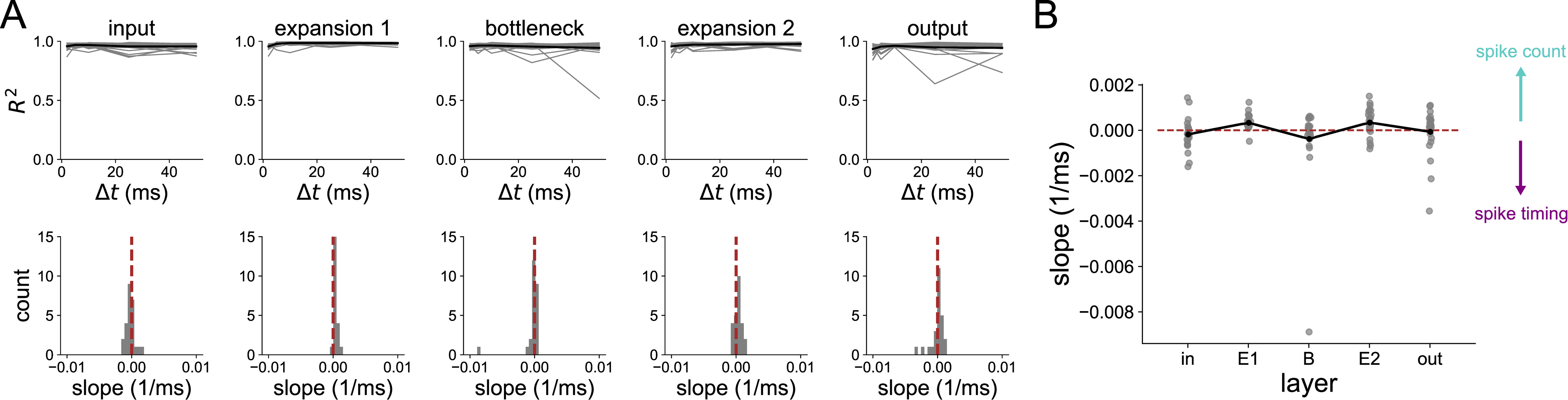

Supplement: S10 Fig — (TIF) [file pcbi.1012971.s010.tif]

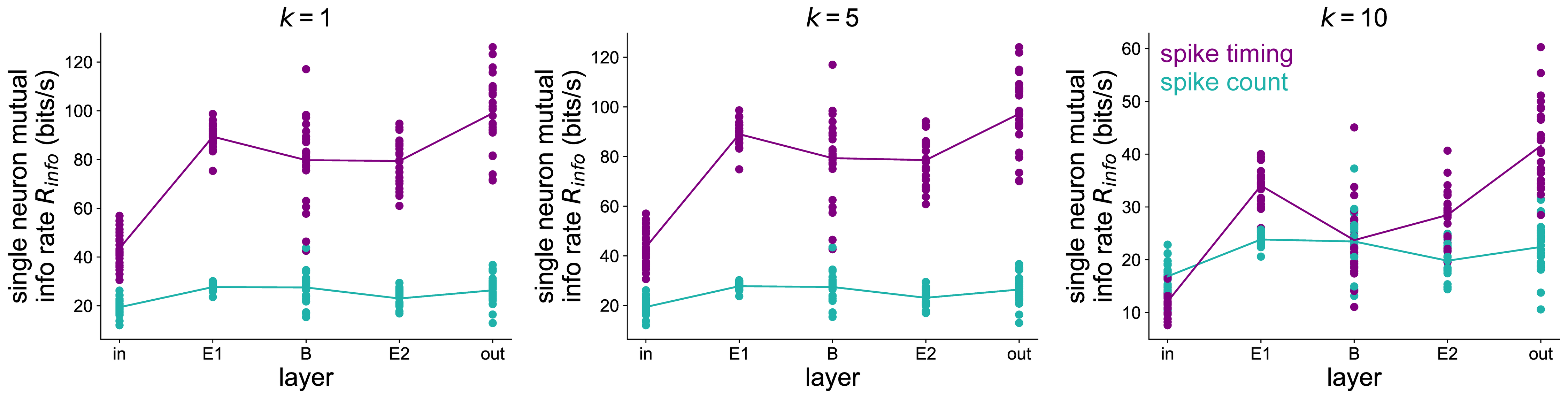

Supplement: S11 Fig — (TIF) [file pcbi.1012971.s011.tif]

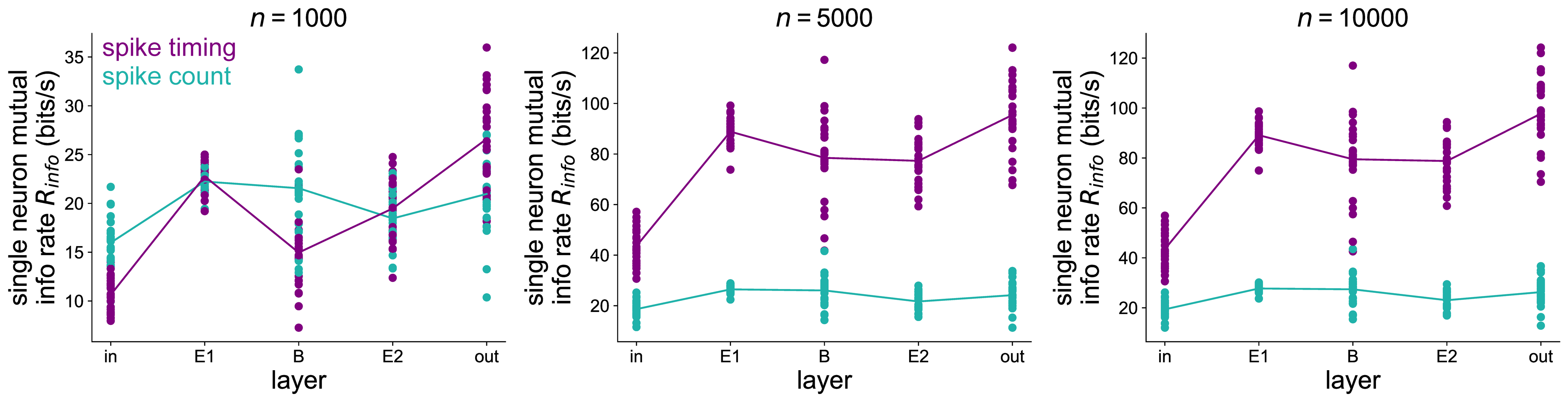

Supplement: S12 Fig — (TIF) [file pcbi.1012971.s012.tif]

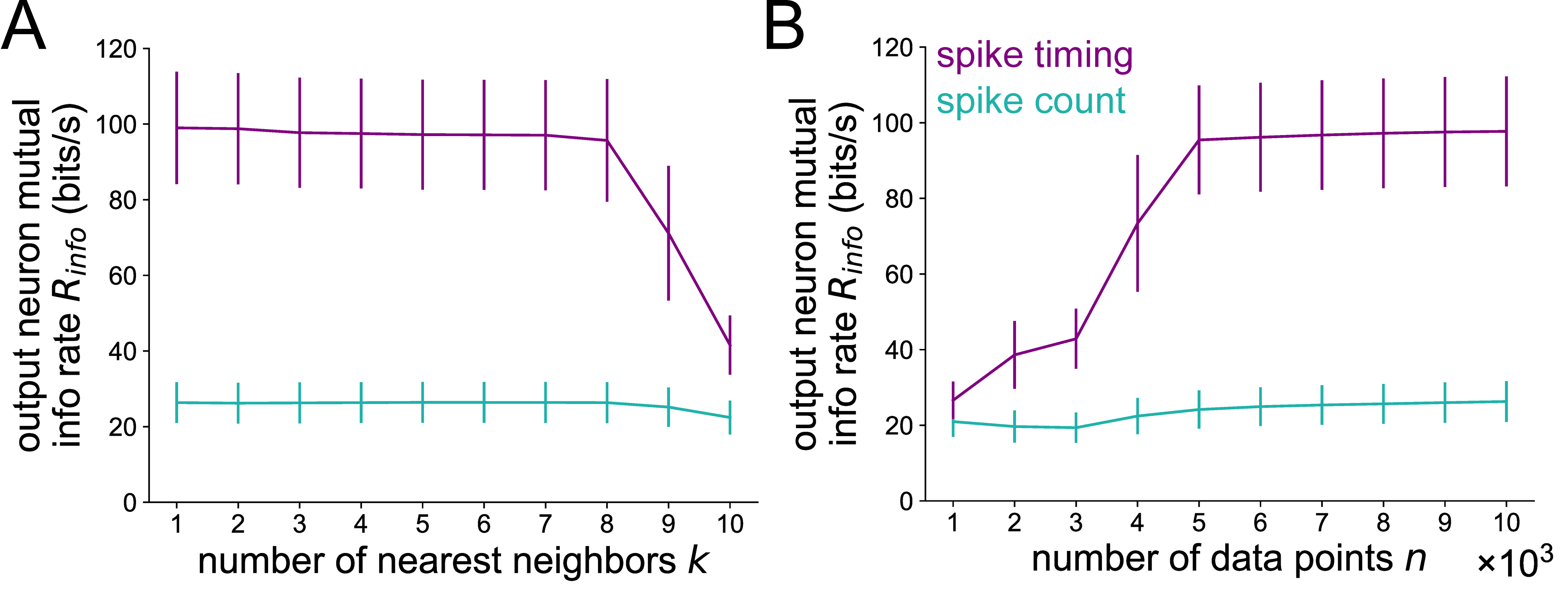

Supplement: S13 Fig — (TIF) [file pcbi.1012971.s013.tif]

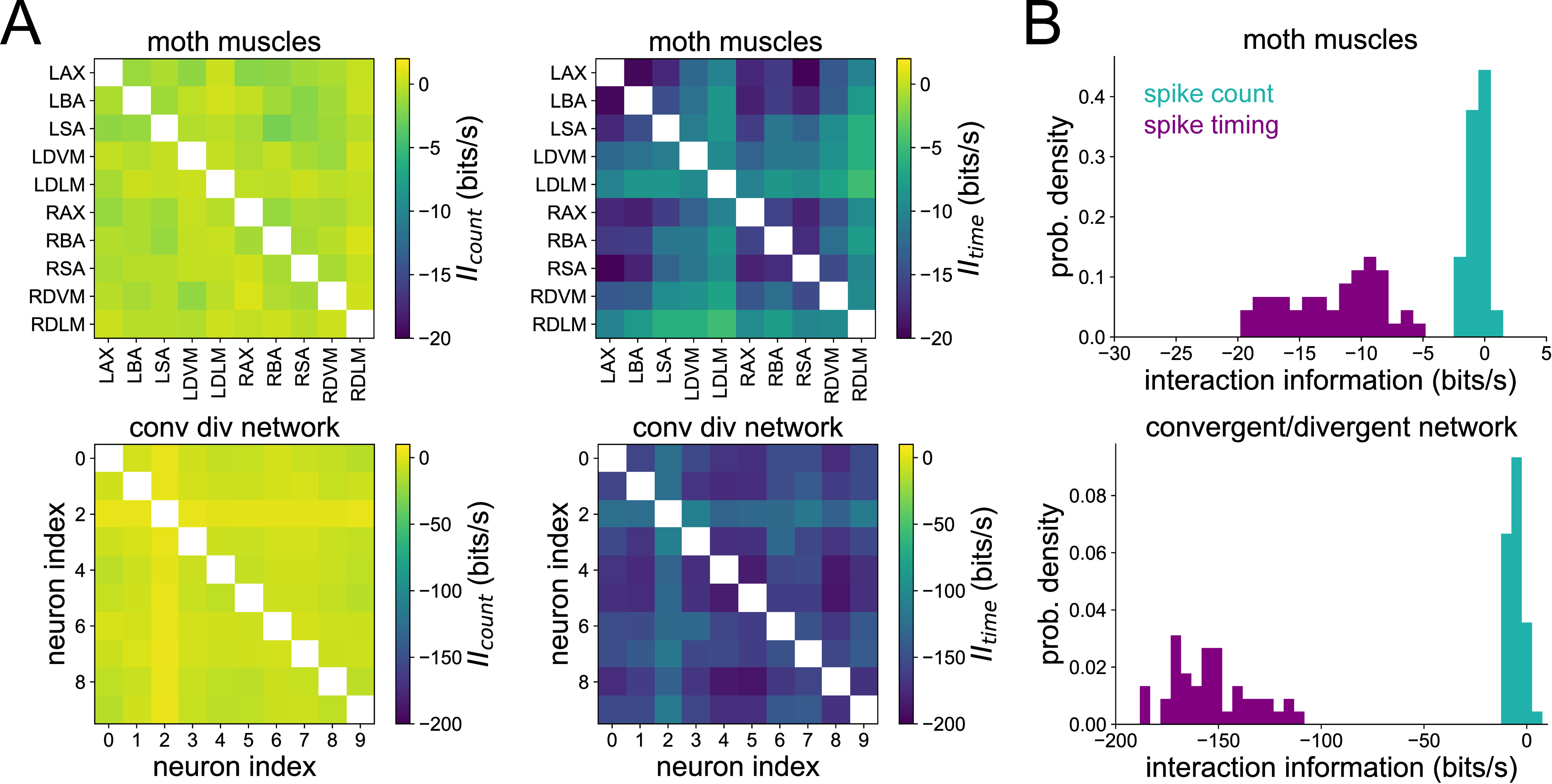

Supplement: S14 Fig — (TIF) [file pcbi.1012971.s014.tif]
