# Supplementary material for: Temporal resolution of spike coding in feedforward networks with signal convergence and divergence
Source: PLoS Comput Biol. 2025 Apr 21;21(4):e1012971. doi: 10.1371/journal.pcbi.1012971 (PMC12021431; doi:10.1371/journal.pcbi.1012971)
Supplement: S1 Appendix — (PDF) [file pcbi.1012971.s015.pdf]

Supporting Information

Generalizability of the three-layer results across spike generators and decoders

Output layer result robustness

This section contains results verifying that our result in Fig 2 is robust to a different spiking (LIF) model and decoder (the gated recurrent unit, GRU).

S1 Fig. Version of Fig 2 but with GRU decoder

S2 Fig. Version of Fig 2 but with LIF spiking model

Hidden layer result robustness

This section contains results that verify the result in Fig 3 is robust to a different spiking model, decoder, and association metric.

S3 Fig. Version of Fig 3 but with mutual information.

S4 Fig. Version of Fig 3 but with GRU decoder.

S5 Fig. Version of Fig 3 but with GRU decoder and mutual information.

S6 Fig. Version of Fig 3 but with LIF neuron model.

Frequency analysis robustness

S7 Fig. Frequency analysis robustness to information metric

Alternative stimuli analysis

S8 Fig. Decoding analysis for sinusoidal stimuli

Robustness to information metric

S9 Fig. All stimuli analysis robustness to information metric

Five-layer model 1 Hz stimulus decoding analysis

This section shows the result of the population decoding analysis from the 5-layer network during a 1 Hz sinusoidal stimulus.

S10 Fig. Decoding analysis of 5 layer model receiving 1 Hz stimulus. (A) Decoding accuracy R^2 between true stimulus s and estimated stimulus $\hat{s}$ from spikes binned at resolution Δt (top) in each layer of the 5-layer model. Each gray line shows the results from one of 25 network seeds; the black curve is the mean. Slopes of the R^2 v.s. Δt curves in each layer (bottom). Vertical line at slope=0 is plotted for reference. (B) Slope distributions versus layer, gray dots are individual network seeds. The black lines connect the means at each layer.

Single-neuron mutual info robustness

This section contains results verifying that the single-neuron mutual information estimates in 5-layer network are robust to variation in the hyperparameter and dataset. In S11 Fig, we show that the single-neuron mutual information estimates across layer are robust to a range of k, the number of nearest neighbors hyperparameter of the KSG method.

S12 Fig shows how the single-neuron mutual information estimates vary with the number of data points used from the output layer of the hawkmoth visuomotor model trained to a 1 Hz sinusoidal stimulus. The general feature where spike count information is similar to spike timing information in the input layer but much lower than spike timing information in the output layer is preserved across data set sizes. The single-neuron information analysis of the output layer is shown against k and n in S13 Fig. Over the ranges of k and n tested, the spike timing information is always significantly higher than the spike count information. In the main results, we use $k = 3$ and $n = 10 \times 10^3$.

S11 Fig. Single-neuron information across layer of the 5-layer model for three values of k, the number of nearest neighbors in the KSG method. Each dot represents the outcome of the single-neuron mutual information analysis for a single 5-layer network seed, pooled across all neurons in the output layer. 25 network seeds used here.

S12 Fig. Single-neuron information across layer of the 5-layer model for various data set sizes n. Each dot represents the outcome of the single-neuron mutual information analysis for a single 5-layer network seed, pooled across all neurons in the output layer. 25 network seeds used here.

S13 Fig. Robustness of the output layer results (A) across number of nearest neighbors k and (B) across data set size n. Error bars represent standard deviations over 25 independent simulations.

Interaction information in hawkmoth model

Another key finding from the previous analysis of Putney et al [25] was that most of the redundancy in the mutual information that moth muscles share with the motor output was in spike timing, not spike count. The authors quantified this through the interaction information between pairs of neurons A and B with motor output m, as defined by

$$II(m; R_A, R_B) = I_m(m; R_A, R_B) - [I_m(m, R_A) + I_m(m; R_B)] \quad (34)$$

where $I_m(m;R_A)$ is the single-neuron mutual information as defined by equation 33 and $I_m(m;R_A,R_B)$ is the joint mutual information that the responses of A and B (R_A and R_B) share with the motor output m, as defined by

$$I_m(m;R_A,R_B) = I_m(m;R_{A,c},R_{B,c}) + \sum_{i_A=1}^{R_{A,c_{max}}} \sum_{i_B=1}^{R_{B,c_{max}}} p(i_A,i_B) I_m(m;R_{A,t},R_{B,t}|(i_A,i_B)) \tag{35}$$

where $R_{A,c}$ and $R_{B,c}$ are the spike counts of neurons A and B, respectively. The maximum spike counts of neurons A and B are $R_{A,c_{max}}$ and $R_{B,c_{max}}$, respectively. The responses $R_{A,t}$ and $R_{B,t}$ are the spike timings of these neurons, which are conditioned on the joint spike count probability distribution $p(i_A,i_B)$. Negative interaction information indicates net redundancy between the pair of neurons. Positive interaction information indicates net synergy. For more details, see ref. [25]. Following Putney et al, we performed this analysis and partitioned the interaction information into spike count and spike timing contributions. We show the results from the original experimental data in the top row of S14 Fig. In the bottom row, we show the result of this analysis performed on the output layer of our 5-layer convergent/divergent network model trained to 1 Hz stimulus. As before, since there is no "motor output" for the model, we use the stimulus s instead of the motor output m in equations 34 and 35, assuming that the motor output reconstructs the stimulus position during tracking. In both the moth data and model data, a majority of the pairwise redundancy is in spike timing instead of spike count, as demonstrated by large negative II_{time} and $II_{count} \approx 0$ in S14 Fig.

S14 Fig. Interaction info in experimental data v.s. 5-layer model. (A) Interaction info from spike count (II_{count}) and spike timing (II_{time}) in experimental data (top) and the output layer of our convergent-divergent 5-layer model of the hawkmoth visuomotor pathway (bottom). (B) Interaction info in spike count and timing pooled across all moth muscles (top) and output layer neurons in the model (bottom).
